# A ten-year report of microbial keratitis in pediatric population under five years in a tertiary eye center

**DOI:** 10.1186/s12348-020-00227-x

**Published:** 2020-11-27

**Authors:** Mohammad Soleimani, Seyed Ali Tabatabaei, S. Saeed Mohammadi, Niloufar Valipour, Arash Mirzaei

**Affiliations:** Ocular Trauma and Emergency Department, Eye Research Center, Farabi eye hospital, Tehran University of Medical Sciences, Tehran, Iran

**Keywords:** Keratitis, Pediatric, Ocular trauma, Children, 5 years, Pediatric, Corneal ulcer

## Abstract

**Purpose:**

To report characteristics of microbial keratitis in pediatric patients under five years.

**Methods:**

Patients with infectious keratitis under the age of 5 years were included in this retrospective cross-sectional study for ten years. All patients were admitted and corneal scraping was performed in 81 children. Fortified empiric antibiotic eye drops including cefazolin (50 mg/cc) and amikacin (20 mg/cc) were started and the antibiotic regimen was continued or changed according to culture results. In the case of fungal keratitis, topical voriconazole (10 mg/cc) or natamycin (50 mg/cc) and topical chloramphenicol (5 mg/cc) were started. A tectonic procedure was done when corneal thinning or perforation was present.

**Results:**

Ninety-Three Patients between 1 to 60 months with a mean age of 33 ± 18 months old with corneal ulcer were included in the study. The most common risk factor was trauma (40.9%) followed by contact lens use (8.6%). Cultures were negative for microbial growth in 28 (30.1%) patients. The most common pathogens were *S. epidermidis* (10.8%) and *P. aeruginosa* (10.8%). Fluoroquinolone antibiotics (ciprofloxacin; 93.8% sensitivity) were the most potent antibiotic against bacterial pathogens. Forty-one patients underwent tectonic procedures, which the most common ones were cyanoacrylate glue 18.3% followed by keratoplasty 16.1%.

**Conclusion:**

This study emphasizes the role of trauma as the primary cause and *S. epidermidis* as the most frequent microorganism in pediatric keratitis; according to antibiogram results and poor cooperation of patients under five years, monotherapy with fluoroquinolones could be a good regimen in small non-central lesions without thinning.

## Introduction

Infectious keratitis is a major global concern due to its morbidity and vision-threatening sequelae especially in developing countries [1]. Although it is rare among pediatric patients, and children only account for about 13% of cases [2, 3], visual deprivation due to decreasing corneal transparency, anisometropia and subsequent amblyopia makes microbial keratitis an important cause of irreversible life-long visual impairment. However, the diagnosis and management are very challenging due to the lack of precise history about the course of the disease and poor cooperation for examination and taking medication [4].

Multiple previous studies all over the world have investigated the prevalence, risk factors and etiology of microbial keratitis in children [3, 5].This study was conducted to investigate epidemiological features of microbial keratitis such as risk factors, the causative organisms, microbial sensitivity, and treatment plans of pediatric patients under five years presenting to a tertiary care ophthalmic center. Up to our knowledge, there is not any study with near one-hundred patients with microbial keratitis of this age group in the literature.

## Patients & methods

This retrospective cross-sectional study took place at Farabi eye hospital, Tehran, Iran as a tertiary ophthalmology care center. Documents of patients were reviewed from January 2008 to December 2017.

This study was approved by the local ethical committee of Farabi Eye Hospital related to Tehran University of Medical Sciences. Patients under the age of 5 years with microbial keratitis were included in this study. Infectious keratitis was defined as a corneal epithelial defect with underlying inflammation due to invasion by bacteria, fungi or acanthamoeba. Patients with isolated suspected viral keratitis (according to the clinical evidence) were excluded from the study.

Ninety-three patients with a corneal ulcer were included in the study. 81 patients underwent an examination under anesthesia (EUA) due to not being cooperative. Some older patients or infants were examined by slit-lamp biomicroscopy. In patients who were examined during EUA, corneal scraping for smear and culture was taken by a cornea specialist with a sterile surgical blade. Patients with simple peripheral lesion,smaller than 3 mm and without thinning did not undergo corneal smear and culture .

Corneal scrapings were sent for gram staining and chocolate, sabouraud, and blood agar plates were used for culture. Susceptibility and resistance tests were done for some patients with antibiogram, if available. All patients were admitted, and fortified empiric antibiotic eye drops including cefazolin (50 mg/cc) and amikacin (20 mg/cc) were started. The antibiotic regimen was continued or changed according to culture results. In the case of fungal keratitis, topical voriconazole (10 mg/cc) or natamycin (50 mg/cc) and topical chloramphenicol (5 mg/cc) were started. Surgical interventions such as glue, amniotic membrane transplantation (AMT), punctal occlusion, blepharorrhaphy, corneal graft (lamellar keratoplasty, penetrating keratoplasty or large corneal graft (graft size> 9 mm)) were performed if needed; large grafts were done in large infiltration, thinning or perforations. Demographic information (age, sex and systemic diseases), clinical characteristics (such as location and size of corneal ulcer, hypopyon, perforation, melting or thinning of cornea), risk factors and causes (including trauma, dry eye, exposure keratopathy, limbal stem cell deficiency, suture abscess, previous *Herpes Simplex Virus* (HSV) infection, contact lens and unknown etiology), previous history of corneal ulcer, tectonic procedures, microbial culture, microbiology analysis and antibiogram were recorded. The incidence of endophthalmitis after the corneal ulcer was also evaluated.

### Statistical methods

Normal distribution of data was assessed by the Kolmogorov–Smirnov test and Q–Q plot. To present data, we used mean, standard deviation (SD), and range. To compare the results between the two groups, we used the t-test, Mann–Whitney test, chi-square test, and Fisher’s exact test. All statistical analyses were performed using SPSS software version 22 (IBM Corp, Armonk, NY). *P* values less than 0.05 were considered statistically significant. All P values are two-sided.

## Results

**Fig. 1:**
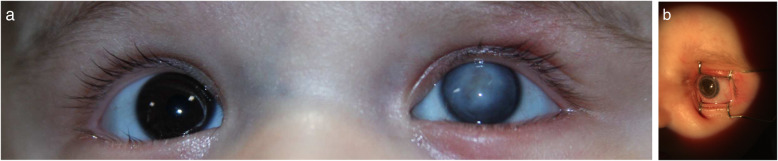
An infant presented with corneal thinning and diffuse infiltration related to *Pseudomonas aeruginosa* keratitis due to nail trauma, the patient underwent amniotic membrane transplantation as a tectonic procedure, **a** shows full thickness corneal scar one month later. **b** shows clear optical graft four weeks after corneal transplantation

Ninety-three patients with a corneal ulcer were included in the study. Patients were between 1 to 60 months with a mean age of 33 ± 18 months old. Fifty-seven patients (61.3%) were male, and 36 of them (38.7%) were female. In more than half of the patients (52.7%), the right eye was involved. Five patients (5.4%) presented with a bilateral corneal ulcer (Fig. 1) (Table 1).

**Table 1:** Descriptive data of pediatric patients with keratitis. OD: Oculus Dexter, OS: Oculus Sinister, OU: Oculus Uterque

Parameter		Value
Age (Month)	Mean ± SD	33 ± 18
	Median (range)	36 (1 to 60)
Sex	Male	57 (61.3%)
	Female	36 (38.7%)
Eye	OD	49 (52.7%)
	OS	39 (41.9%)
	OU	5 (5.4%)

Ulcer size (the largest ulcer diameter) was between 1 to 12 mm (total corneal ulcer) with a mean size of 4.8 mm. Ulcer size was under 2 mm in 21 cases, 2.1–10 mm in 64 cases and larger than 10 mm in 8 cases. There was no correlation between ulcer size and pediatric age (*p* = 0.4). Also, there was not any association between ulcer size and risk of corneal thinning/ perforation (*p* = 0.07). The ulcers were classified as central (central two mm in 48 patients), paracentral (2-6 mm in 6 patients), peripheral (25 patients) or diffuse (involving two or more parts in 9 patients) (according to the most visible extent of the ulcer). No document was available about the location of a corneal ulcer in 5 patients. There was not any association between ulcer location and subsequent tectonic procedure (*p* = 0.1).

Fifteen (16.1%) patients had a history of a previous ulcer which were neither related to current infectious keratitis nor related to any ocular surface diseases. The most common risk factor was trauma (other than chemical injuries) (40.9%) followed by ocular surface disease which were included: contact lens use (8.6% - 8 patients), exposure keratopathy (8.6% - 8 patients) and limbal stem cell deficiency (8.6% - 8 patients) and dry eye (5.4% - 5 patients). Eight patients were using contact lenses (age range: 6 months to four years) including four patients with bandage contact lenses (due to trauma), three patients with soft aphakic contact lens and one patient with hard contact lens. Other predisposing factor was a history of ocular surface disease which were included: shield ulcer (4.3% - 4 patients), history of ocular surface surgery (suture abscess (4.3% - 4 patients), and previous *HSV* infection (2.2% - 2 patients), respectively. The other rare causes were Peters anomaly, progeria, epithelial dystrophy and TORCH syndrome (Toxoplasmosis, Other agents [including *HIV*, syphilis, varicella, and fifth disease], *Rubella, Cytomegalovirus, HSV*). There was no evidence of the associated risk factor in 10 patients. Only two patients had a history of using a topical steroid. (Table 2).

**Table 2:** Causes of pediatric keratitis. LSCD: Limbal stem cell deficiency, HSV: Herpes simplex virus

Causes	Value
Trauma	38 (40.9%)
Ocular surface disease*	29 (31.2%)
Hx of ocular surface disease**	10 (10.8%)
Unknown	10 (10.8%)
Others	6 (6.5%)

* None of the 5 dry eye cases suffer from a systemic disease

** The scars of HSV keratitis were risk factor not the recurrence of the infection

Systemic diseases such as seizure, heart diseases, facial abnormality and diabetes mellitus were seen in 17 patients. Three patients had cerebral palsy. In this study, four patients had a history of retinopathy of prematurity. All of the mentioned patients were referred to the clinic after a recent funduscopic examination.

**Fig. 2:**
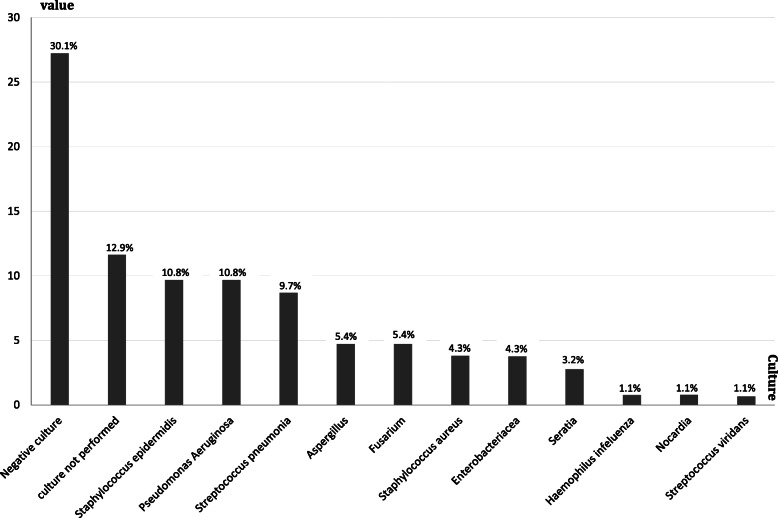
Bar graph illustrates different pathogens in pediatric keratitis

Corneal scraping was performed in 81 children. Cultures were negative for microbial growth in 28 patients (34.5% of this group). The most common pathogens were *S.epidermidis* (10.8%) and *P. aeruginosa* (10.8%) followed by *S. pneumonia* (9.7%). *H. influenza*, *Nocardia, S. viridians* were uncommon organisms (1.1%). *P. aeruginosa* was the most common pathogen in contact lens users. (Table 3, Fig. 2).

**Table 3:** Different pathogens in pediatric keratitis

Cultures	Value*
*S. epidermidis*	10 (10.8%)
*P. aeruginosa*	10 (10.8%)
S. pneumonia	9 (9.7%)
Aspergillus	5 (5.4%)
Fusarium	5 (5.4%)
*S. aureus*	4 (4.3%)
Enterobacteriacea	4 (4.3%)
Seratia	3 (3.2%)
H. influenza	1 (1.1%)
Nocardia	1 (1.1%)
*S. viridans*	1 (1.1%)
Culture not performed	12 (12.9%)
Negative culture	28 (30.1%)

* The value represents the cases not isolated strains

** The rate of methicillin resistant *Staphylococcus aureus* (MRSA) was 0 %

Percentages in this table means the amount of specific organism growth in a culture specimen in relation to whole culture specimens had been taken

Sixteen of 53 culture-positive patients went on to have further evaluation of antibiotic sensitivity and resistance. (Table 4) The results show that fluoroquinolone antibiotics (ciprofloxacin; 93.8% sensitivity) were the most effective antibiotic against bacterial keratitis.

**Table 4:** Sensitivity tests for different usual antibiotics

Amikacin	Sensitive	10	62.5%
	Resistant	5	31.3%
	Not performed	1	6.3%
Cefazolin	Sensitive	8	50.0%
	Resistant	6	37.5%
	Not performed	2	12.5%
Ceftazidime	Sensitive	11	68.8%
	Resistant	4	25.0%
	Not performed	1	6.3%
Vancomycin	Sensitive	7	43.8%
	Resistant	7	43.8%
	Not performed	2	12.5%
Chloramphenicol	Sensitive	9	56.3%
	Resistant	5	31.3%
	Not performed	1	6.3%
	Intermediate	1	6.3%
Gentamicin	Sensitive	11	68.8%
	Resistant	5	31.3%
Levofloxacin	Sensitive	4	25.0%
	Resistant	0	0.0%
	Not performed	12	75.0%
Ciprofloxacin	Sensitive	15	93.8%
	Resistant	0	0.0%
	Intermediate	1	6.3%

Thinning or melting of the corneal ulcer was present in 33 patients and perforation occurred in 20 cases. Forty-one tectonic procedures were done such as cyanoacrylate glue 18.3%, keratoplasty 11.8%, large graft (graft size> 9 mm) 4.3%, and amniotic membrane transplantation 8.6%. Evisceration was done in one patient, because of a severely disorganized eye related to corneal perforation. Post keratitis endophthalmitis (the keratitis that led to endophthalmitis) was found in 2 children leading to pars plana vitrectomy and penetrating keratoplasty.

## Discussion

Despite developments which have been made in past years, microbial keratitis is among one of the major causes of blindness, especially in developing countries which is reported to be almost ten times more than high-income countries [6]. Prevalence of keratitis is less frequent than adults, and it accounts for about 13% of all cases [3]. However, it has a more significant effect on children, because they are prone to amblyopia following visual deprivation and anisometropia which is caused by keratitis. Diagnosis of microbial keratitis in children is also a challenge because of the inability of patients to provide a detailed, accurate history and also the difficulty of slit-lamp examination and preparing smear and culture from the ulcer. Treatment of keratitis in children is another challenge due to poor cooperation for the administration of topical medication.

Almost all previous studies have evaluated keratitis in patients under age of 16 [2, 7–10], but in the current study, we investigated about one hundred children with keratitis under age of 5 which is unique in this way. (Table 5).

**Table 5:** A comparative analysis between previous reports and our report

Reports	Kunimoto DY, et al. 1998	Cruz OA, et al. 1993	Clinch TE, et al. 1994	Ormerod CD, et al. 1986	Hsiao CH, et al. 2007	Rossetto JD, et al. 2017	Eghtedari M,et al. 2018	Song X, et al. 2012	Singh G, et al. 2006	Vajpayee RB, et al. 1999	Our report 2020
Variables											
Most common organism	Staphylococcus 43.7%	*Pseudomonas aeruginosa* 34%	Gram positive cocci 54%	Staphylococcus 34%	Pseudomonas aeruginosa 44.7%	Pseudomonas aeruginosa 46.2%	Staphylococcus 42.8%	Gram positive cocci 41%	Fungus 36.6%	Staphylococcus 70%	Staphylococcus epidermis (10.8%) and Pseudomonas aeruginosa (10.8%)
Principle cause	Trauma 21.2%	Trauma 44%	Trauma 34%	Trauma*	Contact lens wear 40.7%	Contact lens wear 77.6%	Trauma 56.9%	Trauma 58.8%	Trauma 69%	Trauma 38%	Trauma 40.9%
Surgical intervention	15.9%	14%	Not evaluated	28%	14.8%	None	31.6%	77%	9.1%	Not evaluated	44%
Age	Younger than 16 yr	Younger than 16 yr	Younger than 16 yr	Younger than 16 yr	Younger than 16 yr	13 + − 4.6 yr	5.2 + − 4.8 yr	8.9 + −5.7 yr	Younger than 15 yr	4.8 + −3.8 yr	33 + −18 months (1–60 months)

Like most of the previous studies, the most common cause of evolving keratitis is trauma [2, 9–12].The other etiologic factors are contact lens use, exposure keratopathy, and limbal stem cell deficiency. In 2007, Hsiao et al. reported that contact lens use is the most common cause of keratitis in Taiwanese children [3] which is in contrast to our study, probably because of widespread use of contact lenses in that population.

The culture was prepared for 87.1% of these patients. Among these patients, in 69.9% of cultures, bacterial growth was seen. Absence of growth in the other 30.1% of cultures may be due to the use of topical antibiotics before corneal scraping.

**Fig. 3:**
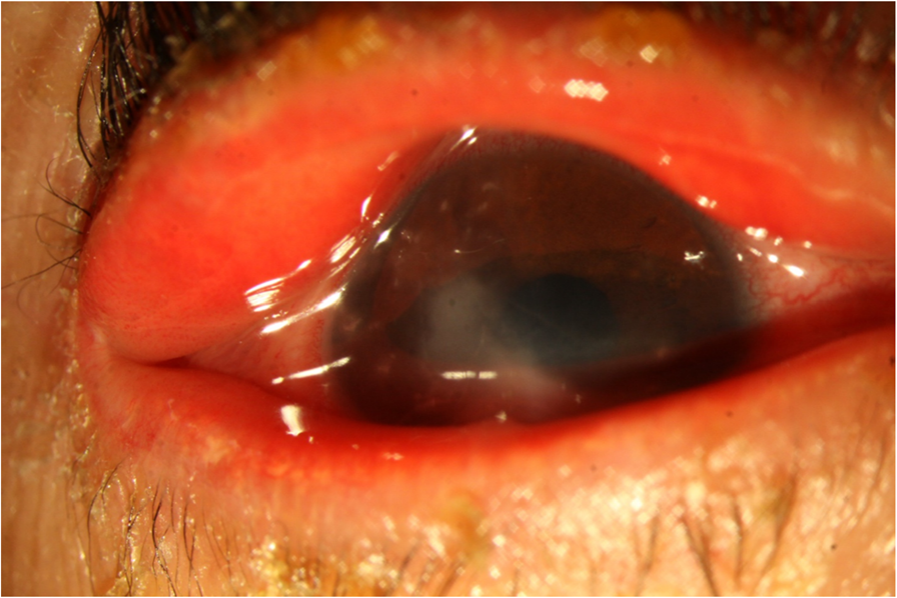
An infant with a S. epidermidis keratitis with a history of ichthyosis

The most frequent bacterial agent responsible for keratitis was found to be *S. epidermidis*, (Fig. 3) and subsequently, *P. aeroginosa* was more common. It seems that contamination with oral microflora is less probable, because, only culture-positive cases were included. In some previous studies [2, 3, 9–11], *P. aeroginosa* was found to be the most common pathogen. This finding may be due to the age difference of studies and therefore, more frequent use of contact lenses in older children. Also, some other studies reported *S. epidermidis* as the most common pathogen [7, 8, 12, 13].Whereas the prevalence of infectious fungal keratitis is 3–37% of all cases in previous studies [3, 8–10], we found that 10.8% of patients were involved with fungal agents.

Unfortunately, several years back, antibiogram was not routinely performed for all microbial cultures in our institute; therefore, we only had access to 16 antibiograms. Analysis of the data on microbial antibiogram showed that 93.8% of agents were sensitive to ciprofloxacin. 62%, 56.3%, and 50% of bacteria were responsive to amikacin, chloramphenicol, and cefazolin, respectively. However, these rates may be affected by small sample size. This analysis could help us determine topical empirical therapy in children under five years of age. Single therapy with ciprofloxacin is by far the best choice for treatment of non-complicated microbial infectious keratitis. Combination of amikacin and chloramphenicol could be the most effective choice for gram-positive bacteria, and administration of amikacin and ceftazidime is the best choice for the eradication of gram-negative bacteria. Jeng and colleagues published a report and mentioned that about 75% of corneal ulcers are responsive to monotherapy with fluoroquinolones [14]. Another study reported no difference in the efficacy of monotherapy with the fourth generation of fluoroquinolones and combination therapy with fortified antibiotic drops in the treatment of microbial corneal ulcer [15]. Since bacteria are still responsive to fluoroquinolones and using a single agent for the treatment of keratitis is more applicable due to less frequent administration of drops, further investigations on safety and efficacy of fluoroquinolones in monotherapy for children is required. Another concern may be related to possible systemic absorption and potential complications of flouroquinolones in pediatric population. However, according to antibiogram results and poor cooperation of patients under five years, monotherapy with fluoroquinolones could be a good regimen in small non-central lesions without thinning.

Forty-one patients underwent surgery with cyanoacrylate glue being the most common procedure followed by tectonic penetrating keratoplasty, amniotic membrane transplantation, pars plana vitrectomy (due to post keratitis endophthalmitis because of echographic and clinical signs of vitreous involvement during or after the diagnosis), and evisceration.

Eghtedari et al. [16] reported 63 cases of keratitis in pediatric patients under 15 years old in Iran. Forty-three patients were under five years old. They concluded that the most common microorganism was *Staphylococcus;* the main predisposing factor was ocular trauma. Although it was similar to our results, they did not perform a separate analysis on under five-year-old patients.

Hsiao et al. [17] also divided pediatric keratitis in 78 children aged 16 years or younger into two groups: group 1 included ages ≤12 years, and group 2 included patients more than 12. They found that the most common cause was trauma and ocular disease in the group 1 despite contact lens wear in group 2.

There are some drawbacks to this study. The retrospective nature of the study made it prone to information bias. Lack of proper and detailed documentation in the past years, and performing antibiogram on only 16 cultures are other limitations of the study. Another limitation is that data from the patients who were referred to a tertiary center and had more difficult and recalcitrant conditions. Because of retospective nature, we did not have access to the percent of patients suffered from amblyopia.

However, despite these limitations, this study is the first of its kind which was done on this number of patients under the age of five.

## Conclusion

This study shows the role of trauma as the primary cause of pediatric keratitis, *S. epidermidis* was the most frequent microorganism in pediatric keratitis. Monotherapy with fluoroquinolones could be a good option in small non-central lesions without thinning, because of the ease of usage in children under five years.

## Data Availability

The datasets used and/or analysed during the current study are available from the corresponding author on reasonable request.

## Abbreviations

AMT: Amniotic membrane transplantation
EUA: Examination under anesthesia
SD: Standard deviation
HSV: Herpes simplex virus
TORCH syndrome: Toxoplasmosis, Other agents [including *HIV*, syphilis, varicella, and fifth disease], *Rubella, Cytomegalovirus, HSV*
